# Biological markers of hearing loss in neonates admitted to the neonatal intensive care unit: a systematic review and meta-analysis

**DOI:** 10.3389/fnins.2026.1796635

**Published:** 2026-03-31

**Authors:** Sally K. Thornton, Roshni Patel, Sandra Smith, Sara Ahmadinejad Farsangi, Helen Brough, Mohamad Amin Pourhoseingholi, Dulip Jayasinghe, Derek James Hoare

**Affiliations:** 1 Hearing Sciences, Mental Health and Clinical Neurosciences, School of Medicine, University of Nottingham, Nottingham, United Kingdom; 2 NIHR Nottingham Biomedical Research Centre, Nottingham, United Kingdom; 3 Children’s Audiology, Nottingham University Hospitals, Nottingham, United Kingdom; 4 Neonatal Intensive Care Unit, City Hospital Campus, Nottingham University Hospitals, Nottingham, United Kingdom

**Keywords:** bilirubin, serum biomarker, hearing loss, infection, metabolic, neonate, neurodevelopmental impairment, NICU (neonatal intensive care unit)

## Abstract

**Background:**

Infants admitted to neonatal intensive care units are at increased risk of hearing loss, yet early identification remains challenging. Understanding circulating biomarkers of hearing loss may improve risk stratification and inform targeted surveillance and intervention.

**Objectives:**

To assess whether biological factors measured in bodily fluids predict hearing loss in neonates admitted to the neonatal intensive care.

**Methods:**

Systematic review and meta-analyses were conducted (registration ID: CRD42024531492). Comprehensive searches were undertaken to identify peer-reviewed studies published between 2000 and 2025 evaluating fluid biomarkers and hearing outcomes in neonates. Risk of bias was assessed across five domains. Biomarkers were grouped by biological domain and synthesised narratively. Where feasible, random-effects meta-analyses were performed using odds ratios and restricted maximum likelihood estimation for calculation of pooled effect sizes.

**Results:**

Eighty-six studies were included, predominantly retrospective cohorts with substantial methodological heterogeneity. Elevated bilirubin exposure (*n* = 44 studies) was consistently associated with hearing loss, with unbound bilirubin demonstrating superior predictive performance. Infective conditions (*n* = 32 studies), particularly congenital cytomegalovirus, meningitis, and invasive fungal infections showed significant associations with hearing loss. Risk of bias was moderate to high across studies, driven by inconsistent hearing outcomes measures, poor repeatability of biomarker measures and limited inclusion of risk factors and confounders which affect biomarker concentrations.

**Discussion:**

Systemic neonatal biomarkers, particularly unbound bilirubin and infective markers, show consistent associations with hearing loss, though high heterogeneity limits precision. Evidence was heterogeneous and largely exploratory. Future studies should utilise integrated databases where data have rigorous confounder adjustment, standardised biomarker assessments, and validated hearing outcome measures to identify clinically meaningful biomarkers of hearing loss.

**Systematic review registration:**

https://www.crd.york.ac.uk/PROSPERO/, identifier CRD42024531492.

## 1 Introduction

There is a high risk of hearing loss among neonates admitted to the neonatal intensive care unit (NICU). Hearing loss is a clinically important morbidity with long-term consequences for language development, communication, cognition, and educational outcomes (Chant et al., 2022; Coenraad et al., 2010; Moosan et al., 2024). Although universal newborn hearing screening (UNHS) has improved detection diagnostic pathways (Harrop-Griffiths, 2016) they are usually initiated after infants are medically stable or discharged from intensive care. As a result, early biological indicators of hearing loss present during critical illness may be overlooked. Identifying biological markers associated with hearing loss during NICU admission could improve early risk stratification, guide follow-up, and inform future preventive or neuroprotective strategies.

A body-fluid biomarker of hearing loss can be defined as a measurable molecular entity in blood, cerebrospinal fluid (CSF), or other biological fluid, that reflect pathological processes affecting the auditory system. For clinical relevance, biomarkers must demonstrate biological plausibility, analytical validity, and consistent associations with hearing outcomes such as degree of hearing loss. Many clinically important serum and CSF biomarkers are routinely recorded on the NICU. While numerous candidate biomarkers reflecting neurotoxicity, inflammation, hypoxia, and systemic illness have been reported in NICU populations, their relative predictive value for hearing outcomes remains unclear. This uncertainty highlights the need to better leverage clinically available biomarkers that could translate research findings into meaningful prognostic tools.

Bilirubin is the most extensively studied biomarker of neonatal hearing loss (Akinpelu et al., 2013; Merino-Andrés et al., 2024; Olds and Oghalai, 2015) and neurodevelopmental impairment (Wusthoff and Loe, 2015). Hearing loss associated with bilirubin is often called auditory toxicity (AT) as it reflects damage caused by unbound bilirubin (UB)—resulting in sensorineural hearing loss (SNHL) and/or auditory neuropathy spectrum disorder (ANSD). ANSD is a type of sensorineural hearing loss characterised by a mismatch where the outer hair cells of the cochlea appear to function normally, while the inner hair cells or auditory nerve and/or the synaptic connections between the two do not function reliably. Hyperbilirubinaemia (HB) can injure cochlear hair cells and auditory neurons, and hearing loss is a recognised feature of acute bilirubin encephalopathy (ABE) and chronic bilirubin encephalopathy (CBE) latterly known as kernicterus. ABE represents the early, potentially reversible neurotoxic effects of severe unconjugated HB on the neonatal brain, whereas CBE reflects permanent injury, particularly to the auditory brainstem and basal ganglia resulting in long-term sensorineural hearing loss and ANSD.

Total serum bilirubin (TSB) is a widely used measure in clinical practice. However, its specificity for predicting hearing loss is poor, depending on infant population (stability, gestational age etc.) with wide variability in reported rates of hearing loss among exposed infants (Amin et al., 2016). Unbound (free) bilirubin and the bilirubin-to-albumin ratio are biologically plausible alternatives and may better reflect neurotoxic risk. Yet, they are not routinely measured and evidence supporting their superiority remains fragmented.

Importantly, bilirubin-associated hearing loss is strongly modified by systemic illness. Coexisting conditions such as sepsis, haemolysis, metabolic acidosis, prematurity, and genetic vulnerability lower the threshold for neurotoxicity (Gamaleldin et al., 2011; Singh et al., 2021). Infection itself is a major cause of neonatal hearing loss, particularly congenital cytomegalovirus infection (cCMV) (Zhang et al., 2025), bacterial meningitis (Sharma et al., 2022), and invasive fungal disease [ Weimer et al., 2022). Although inflammatory and infection-related biomarkers (e.g., C-reactive protein (CRP), interleukin-6 (IL-6), procalcitonin, and CSF indices] are widely used for diagnosis and disease severity assessment, their prognostic value for hearing outcomes has not been systematically synthesised, with existing evidence largely derived from heterogeneous, condition-specific cohorts.

Other biological pathways implicated in hearing loss, including hypoxia, metabolic disturbance, and multi-organ dysfunction are understudied. Many of these factors are not included in current neonatal hearing surveillance frameworks, reflecting a broader gap in the evidence base (Joint Committee on Infant Hearing [JCIH], 2019; NHSP, 2022). To date, no comprehensive synthesis has evaluated body-fluid biomarkers associated with hearing loss across NICU populations, or their predictive performance.

The primary focus of this review is on biomarkers associated with hearing loss; however, many NICU cohort studies report hearing outcomes within composite measures of NDI. Where hearing loss was included as a defined component of a broader neurological outcome, such studies were considered in order to capture the full scope of available biomarker evidence.

The primary aim of this systematic review and meta-analysis was to synthesise evidence on body fluid biomarkers measured during NICU admission that may be associated with subsequent hearing loss. The objectives were to identify and categorise relevant biomarkers, evaluate the strength and consistency of their associations with hearing outcomes, compare biomarker measures where possible, examine the modifying role of systemic illness, and identify gaps to inform future research and thus clinical practice.

## 2 Materials and methods

### 2.1 Registration

This review was pre-registered on PROSPERO registration number: CRD42024531492.

### 2.2 Studies and participants

Studies were eligible if they evaluated fluid biomarkers in relation to hearing loss. Given the structure of much of the neonatal literature, studies reporting composite outcomes (e.g., neurodevelopmental impairment) were included if hearing loss was explicitly defined as a component outcome or if hearing-specific data could be extracted.

Biomarkers were extracted as reported in the original studies and were categorised as either quantitative measures (e.g., peak serum levels) or binary clinical exposures (e.g., presence of meningitis), reflecting the heterogeneity of biomarker operationalisation in the neonatal literature.

#### 2.2.1 Type of studies

Investigational or exploratory studies, basic science and clinical research studies from the year 2000 until November 2025. The year 2000 was chosen as the early 2000’s marked the rollout of newborn hearing screening in many countries.

#### 2.2.2 Participants

Inclusion: Human neonates admitted to intensive care, hearing loss.

Exclusion: Animal studies, adults, history of oncology and chemotherapy, syndromes and genetics.

### 2.3 Search terms and databases

A systematic search strategy was employed to identify relevant articles from literature search platforms; EMBASE, ASSIA, PubMed and EBSCO Host, using the search terms: neonat* AND (hearing* OR deaf*) AND (marker OR protein OR immunoglobulin OR enzyme OR cytokine OR interleukin OR glucose OR lactate* OR blood pH OR base excess OR troponin OR creatine* OR sepsis OR hypoxi*).

**FIGURE 1:**
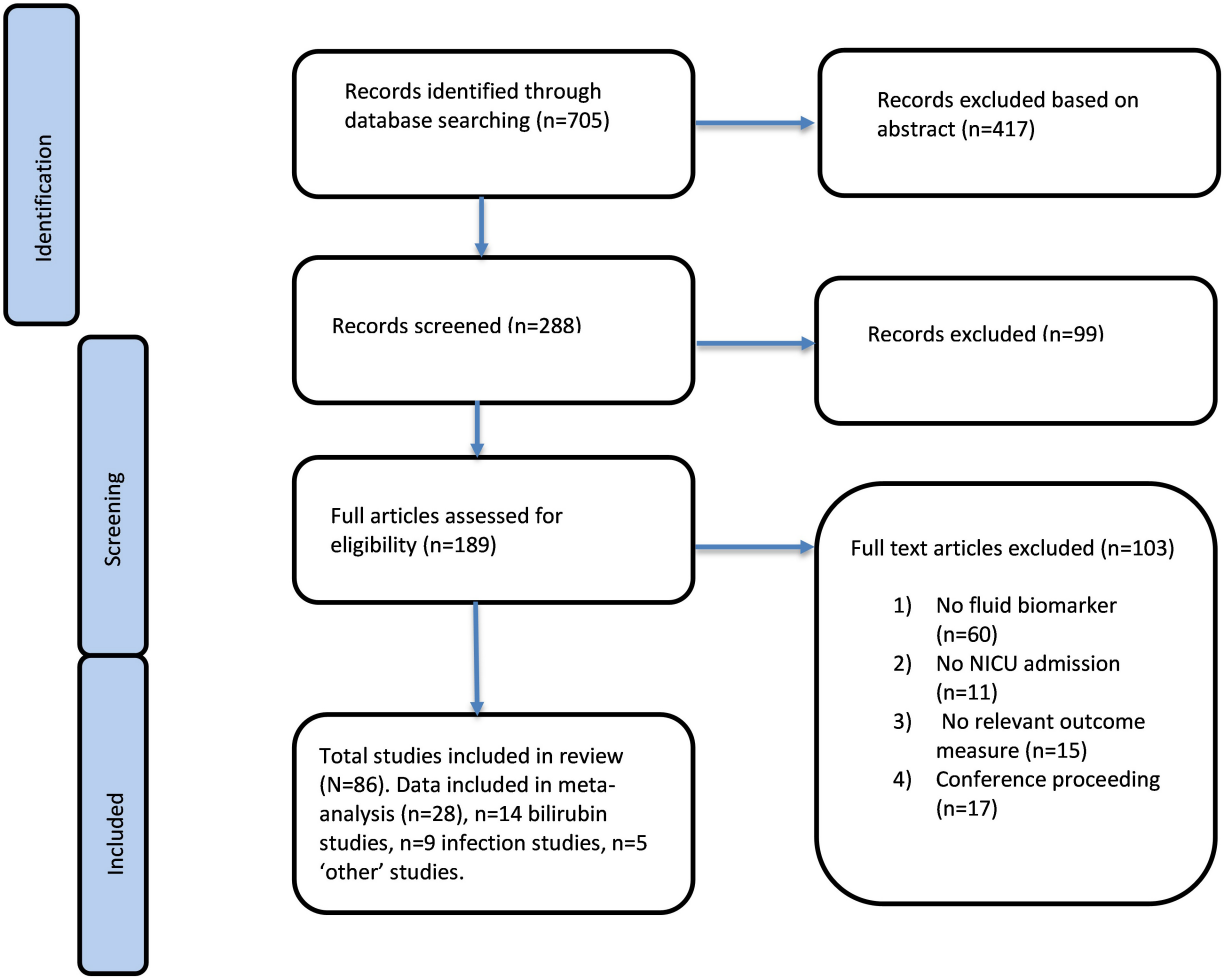
Reported items for systematic review and flow diagram of record screening and selected studies. Flow diagram illustrating study selection—the identification, screening, eligibility assessment, and inclusion of studies in accordance with the PRISMA 2020 guidelines, including records identified through database searching and other sources, records excluded at each stage, and the final number of studies included in the systematic review.

See Figure 1 for flow chart summarising record screening of selected studies.

The initial search (August 2023) was complemented by scanning reference lists from relevant systematic reviews and the included primary studies; citation searching of the included primary studies using Web of Science; and hand searching the last 6 months of key audiology and ENT journals. Searches were limited to articles in the English language published in peer reviewed journals. Update searches were conducted in July 2024, January 2025 with the final update search conducted in November 2025.

### 2.4 Selection of studies

Records were screened independently by two members of the review team, first by titles and abstract, and then by full text where the abstract indicated the record was likely relevant or there was insufficient information in the abstract to decide.

### 2.5 Data extraction and management

**FIGURE 2:**
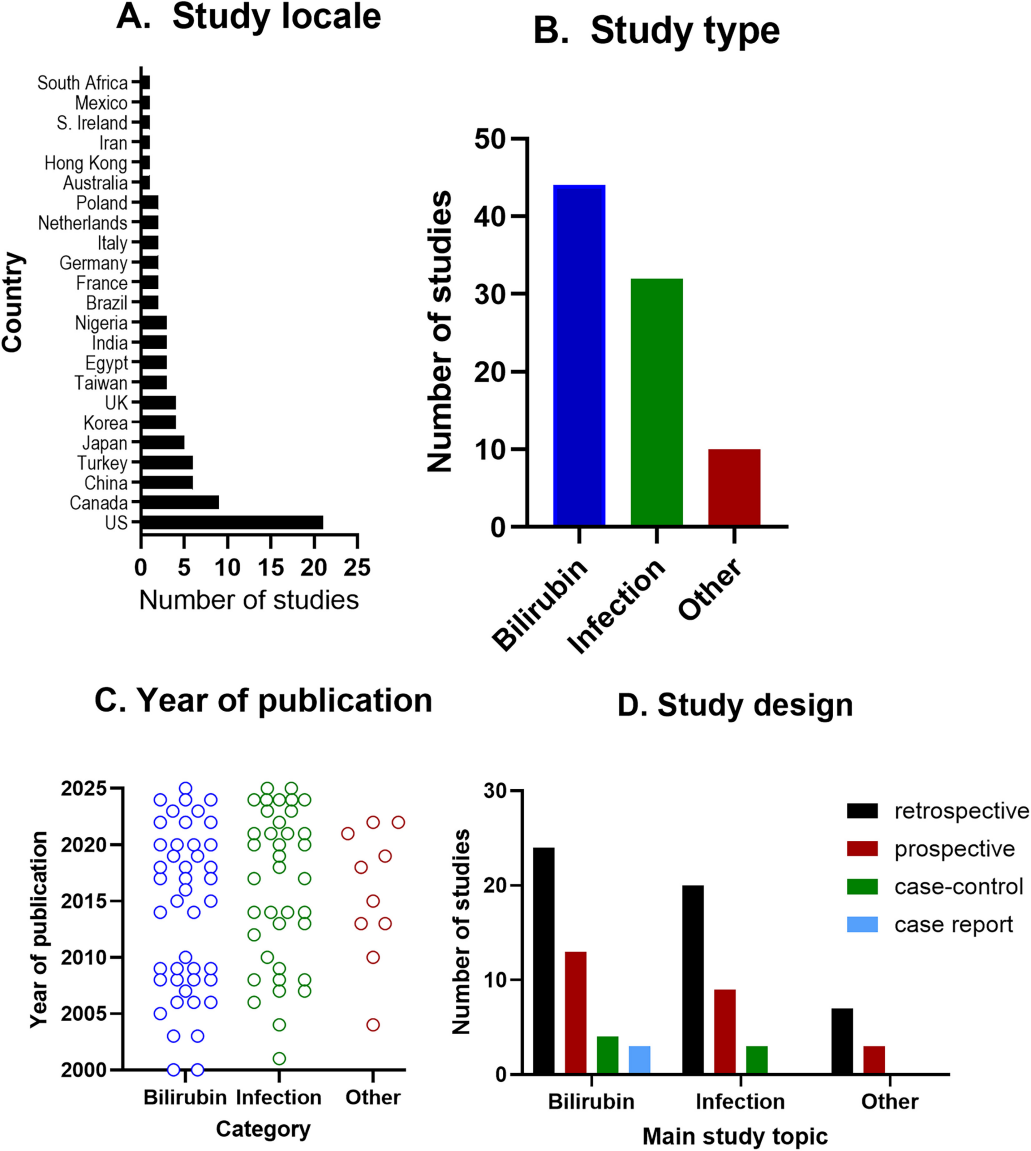
Descriptive study information - illustrating the contextual and methodological characteristics of studies included in the systematic review. Contextual and methodological characteristics of included studies. This figure summarises the descriptive features of studies included in the systematic review, including **(A)** geographical distribution by country, **(B)** primary study topic (bilirubin-related, infection-related, or other biomarkers), **(C)** year of publication stratified by study topic, and **(D)** study design across main topics (retrospective, prospective, case–control, and case report).

For included studies, data were extracted independently by three members of the review team (two for each record) using a data extraction form which was developed for purpose and piloted prior to its use. Any disagreements in the extracted data were resolved through discussion or consultation with a fourth member of the review team. Extracted data included participant information (demographics, baseline characteristics, sample size), country, study design, research questions, hearing and health characteristics and biological factor(s) assessed, key data, findings and conclusions (see Figure 2 and the summary data on each biomarker can be found in Supplementary files).

### 2.6 Risk of bias (quality) assessment

**FIGURE 3:**
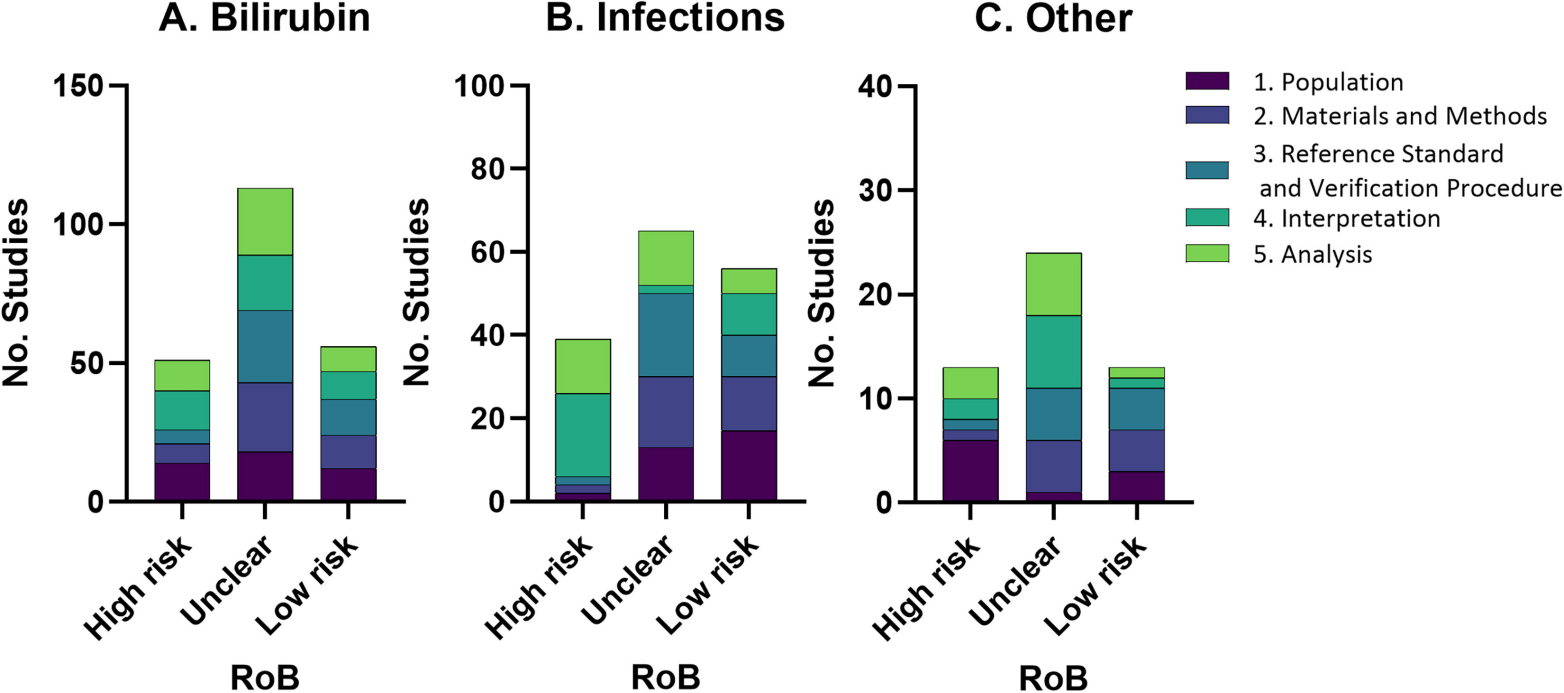
Risk of bias (RoB) for bilirubin **(A)**, infections **(B)** and “other” **(C)** studies across five domains. Risk of bias (RoB) assessment assessed five key categories of medical test performance (Santaguida et al., 2012). Records assessed (1) Population bias (spectrum effects, context or selection bias), (2) Test protocol (variations in test execution or test technology), (3) Reference standard and verification (inappropriate reference standard, differential or partial verification bias), (4) Interpretation (review or incorporation bias, observer variability), and (5) Analysis (handling of indeterminate results, arbitrary choice of threshold values).

Risk of bias assessment assessed five key categories of medical test performance as previously reported (Santaguida et al., 2012). Records assessed (1) Population bias (spectrum effects, context or selection bias), (2) Test protocol (variations in test execution or test technology), (3) Reference standard and verification (inappropriate reference standard, differential or partial verification bias), (4) Interpretation (review or incorporation bias, observer variability), and (5) Analysis (handling of indeterminate results, arbitrary choice of threshold values). For each category a quality judgment was made, scored as either “Yes” (low risk of bias), “No” (high risk of bias), or “Unclear” (insufficient information in the record to make a judgment). When rating a study “No” on any domain the reviewer will note the reasons why. Two reviewers independently assessed risk of bias within each record. A third reviewer arbitrated when required. Data from the risk of bias assessments across studies was summarised in a descriptive narrative according to the five categories of medical test performance (Figure 3).

**FIGURE 4:**
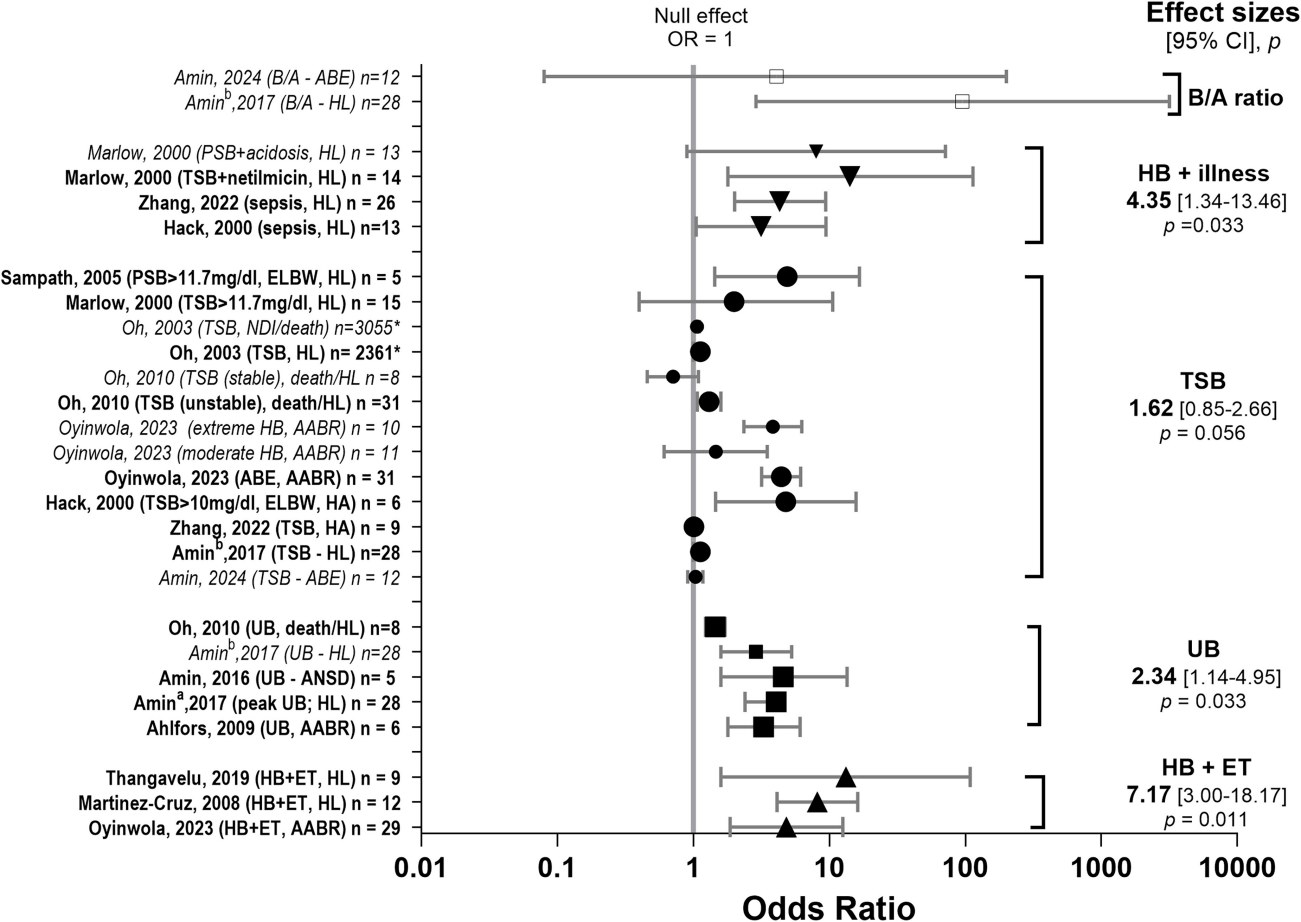
Effect sizes for bilirubin biomarkers. Associations between bilirubin biomarkers and hearing loss. Forest plot summarising pooled odds ratios (ORs) and 95% confidence intervals (CIs) for the association between bilirubin-related biomarkers and hearing loss across studies. Individual study estimates are displayed with corresponding 95% CIs, and pooled effect estimates are shown for each biomarker category, including bilirubin/albumin (B/A) ratio □ (35,59), hyperbilirubinaemia (HB) in the presence of clinical illness (HB + illness) ▼(36,37,44), total serum bilirubin (TSB) (28,34–38,43,44,102) ●, unbound bilirubin (UB) ■ (9,34,35,38,46), HB and exchange transfusion (HB + ET) ▲(41,43,45). The vertical line at OR = 1 represents the null effect. ORs greater than 1 indicate increased odds of hearing loss. Pooled estimates were calculated using random-effects meta-analysis. The studies contributing to each pooled estimate are indicated by emboldened text and enlarged symbols in the figure. AABR, automated auditory brainstem response; ABE, acute bilirubin encephalopathy; ANSD, auditory neuropathy spectrum disorder; B/A, bilirubin/albumin ratio; ELBW, extremely low birth weight; ET, exchange transfusion; HB, hyperbilirubinaemia; HL, hearing loss; HA, hearing aid; NDI, neurodevelopmental impairment; PSB, peak serum bilirubin; TSB, total serum bilirubin; UB, unbound bilirubin.

**FIGURE 5:**
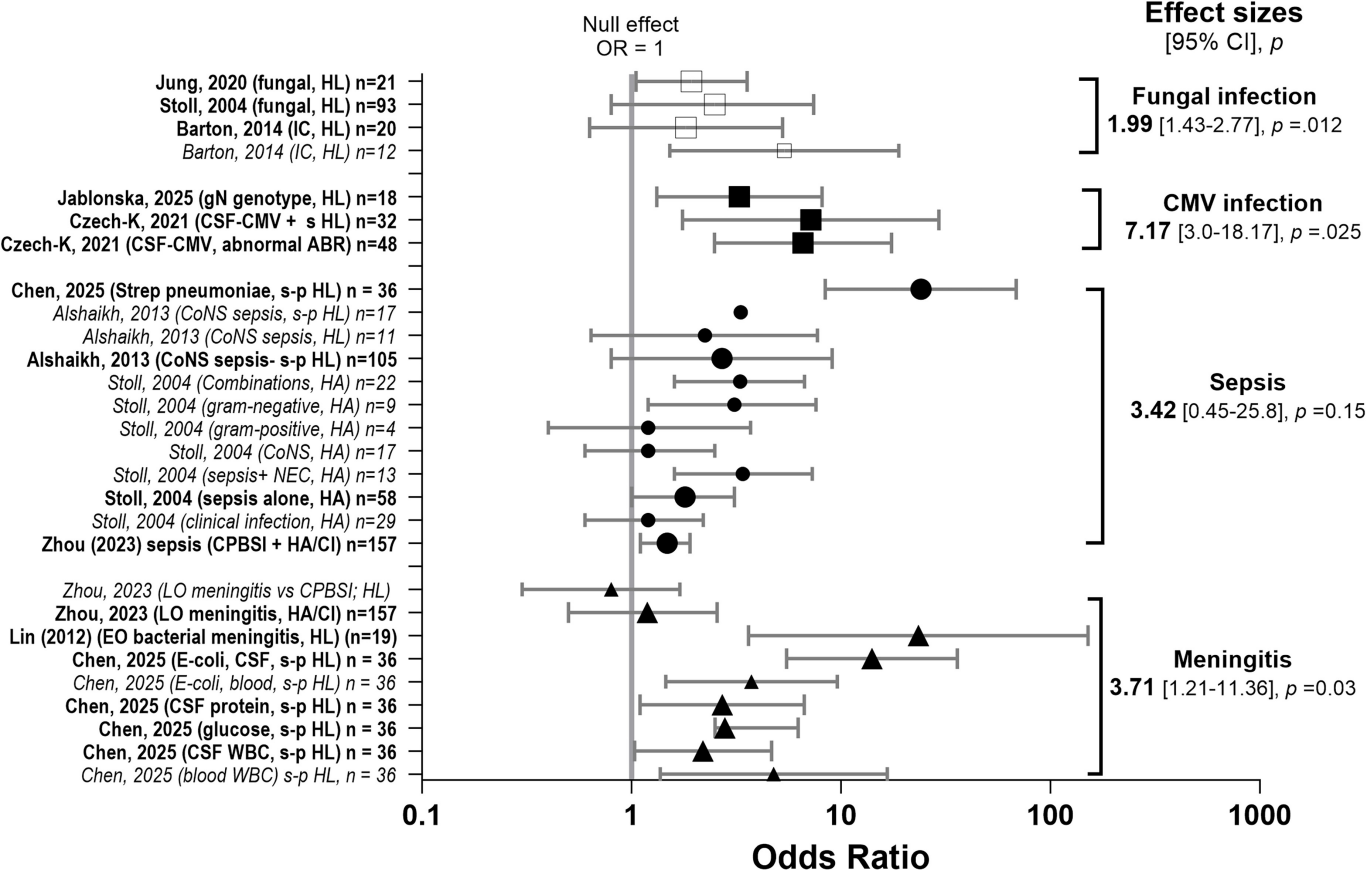
Effect sizes for infection biomarkers. Associations between infection-related biomarkers and hearing loss. Forest plot summarising pooled odds ratios (ORs) and 95% confidence intervals [CI] for the association between infection-related biomarkers and hearing loss across studies. Individual study estimates are displayed with corresponding 95% CIs, and pooled effect estimates are shown for each biomarker category, including fungal infection □ (70,75,78), cytomegalovirus (CMV) infection ■ (73,88), sepsis infection ● (60,75,87,107), meningitis▲ (60,61,87). The vertical line at OR = 1 represents the null effect. ORs greater than 1 indicate increased odds of hearing loss. Pooled estimates were calculated using random-effects meta-analysis. The studies contributing to each pooled estimate are indicated by emboldened text and enlarged symbols in the figure. CSF, cerebrospinal fluid; CoNS, coagulase-negative staphylococcus; CPBSI, culture-positive bloodstream infection; CMV, cytomegalovirus; HA, hearing aid; HA/CI, hearing aid or cochlear-implant, HL, hearing loss; s HL, severe hearing loss; s-p HL, severe-profound HL; IC, invasive candidiasis; LO, late-onset; necrotising enterocolitis, NEC; WBC, white blood cells.

**FIGURE 6:**
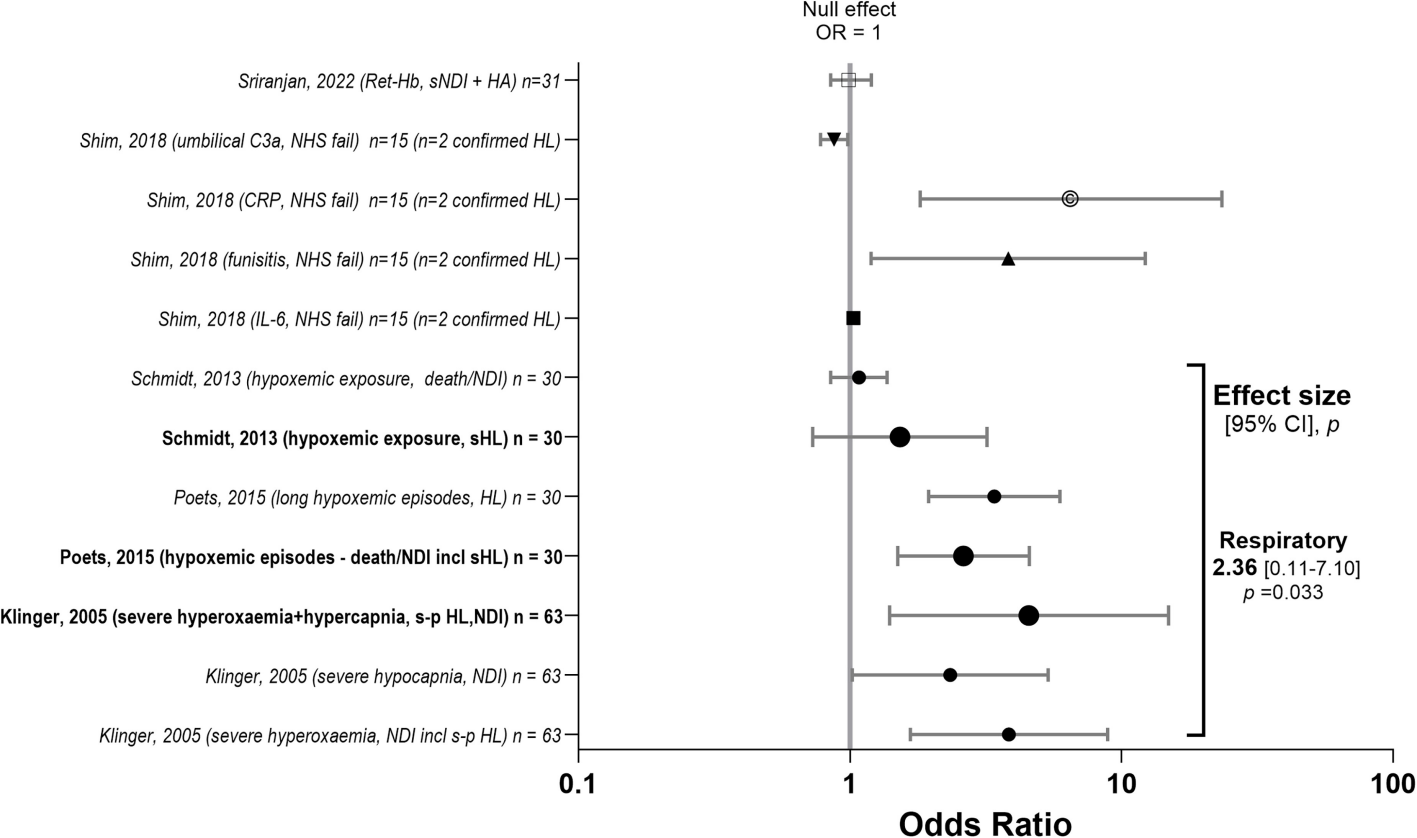
Effect sizes for “other” biomarkers. Associations between “other” biomarkers and hearing loss. Forest plot summarising pooled odds ratios (ORs) and 95% confidence intervals (CI) for the association between “other” biomarkers and hearing loss across studies. Individual study estimates are displayed with corresponding 95% CIs, and pooled effect estimates are shown for each biomarker category, including haematological (iron) □ (91), umbilical C3a ▼(96), C-reactive protein (CRP) ©(96), funisitis ▲(96), interleukin-6 (IL-6) ■ (96) and respiratory ● (97–99). The vertical line at OR = 1 represents the null effect. ORs greater than 1 indicate increased odds of hearing loss. Pooled estimates were calculated using random-effects meta-analysis. The studies contributing to each pooled estimate are indicated by emboldened text and enlarged symbols in the figure. C3a, complement C3a (peptide in umbilical cord); CRP, C-reactive protein; CSF, cerebrospinal fluid; HA, hearing aid; HA/CI, hearing aid or cochlear-implant, HL, hearing loss; s HL, severe hearing loss; s-p HL, severe-profound HL; interleukin-6, IL-6; IC, invasive candidiasis; LO, late-onset; neurodevelopmental impairment, NDI; UNHS, universal newborn hearing screening; ret-Hb, reticulocyte-haemoglobin (measures functional iron available for erythropoiesis).

Hearing outcome measures were also affected by bias. For example, AABRs and referral from the UNHS can reflect immaturity and does not necessarily equate to confirmed permanent hearing loss. There was only a small subset of studies where hearing screening referral or AABR results were used as the primary outcome measure. These are labelled as AABR in Figure 4 and UNHS refer in Figure 6. UNHS in Figure 5 refers to a test which includes AABR and otoacoustic emissions.

### 2.7 Data analysis

Analyses were conducted by a single reviewer (SKT) and checked for accuracy by a second reviewer. Records were grouped according to which biological variable(s) they measured, and each biomarker was analysed separately. Data from individual studies were reported descriptively—mean values from individual measures, and correlations between biological factors and presence or severity of hearing loss within studies, were reported. The majority of the studies did not assess biomarkers of hearing loss as the primary aim, so it was rare for receiver operator characteristic (ROC) curves or sensitivity/specificity of the biomarker to be recorded. Binary outcomes data were predominately presented as number of children with (out) HB/infection/other (or number of neonates with high/low concentrations exceeding a defined threshold or treatment level) and hearing loss reported as an outcome measure, often as part of the reporting of neurodevelopmental impairment (NDI). Thus, odds ratios (OR) were most often reported. Dichotomous outcomes were expressed as OR and 95% confidence intervals (CI). The findings from the OR reveal the strength and direction of association between an exposure (e.g., TSB or infection) and an outcome (hearing loss or NDI, with OR > 1 indicating increased odds (risk factor), OR < 1 indicating decreased odds (protective factor), and OR = 1 indicating no association; for statistical significance, the 95% confidence interval must not include 1. No data from case reports were included in the meta-analysis.

#### 2.7.1 Statistical analyses

Statistical analyses were completed in accordance with PRISMA 2020—Statistical Methods (Item 13). Meta-analyses were performed using random-effects models to account for between-study heterogeneity. Effect estimates were pooled using inverse-variance weighting and reported with 95% confidence intervals. The statistical significance of the overall pooled effect was assessed using a Z test. Between-study variance was estimated using the restricted maximum likelihood (REML) method. Effect sizes were pooled using inverse-variance weighting and are reported with 95% confidence intervals (CI).

Statistical heterogeneity was assessed using Cochran’s Q test and quantified using the *I*^2^ statistic. Substantial heterogeneity was anticipated due to variability in neonatal populations, biomarker measurement protocols, outcome definitions, and study designs. Heterogeneity was high so random-effects models were used, and findings were interpreted cautiously. Overall effect size was reported in text, subgroup effect sizes were reported in forest plots.

For each biomarker the OR was reported for individual studies (Figures 4–6). The SE and LnOR were pre-calculated from the source data where SE = (Ln (Upper 95% CI)-Ln (Lower95% CI))/3.92. All data were calculated in natural Ln format but log_10_ OR were plotted on the x-axes of the forest plots as this is more conventional for forest plots.

Following the results of high heterogeneity we subdivided the biomarkers in each group. When studies reported multiple ORs derived from the same participant sample, we reduced the independent effect estimates to only be included in the meta-analysis if they had unrelated outcome measures, to avoid multiplicity bias and statistical dependency. Additional effect estimates were excluded from the pooled effect size calculation in accordance with PRISMA 2020 guidance on handling dependent data. However, since the biomarker data were exploratory in nature, and the research was not at the stage to inform clinical guidelines we included more than one study per source—if the assay results were independent measures or if they were measured on different populations (López-López et al., 2018). The choice of biomarkers to include was also dictated by clinical stewardship (neonatal consultant). If there was a choice to be made and all parameters were equivalent, then data containing the larger sample size were included in the pooled effect size calculation.

Potential publication bias was assessed through visual inspection of funnel plot asymmetry (Supplementary Figure 1). Risk of bias was assessed using Egger’s regression test within a random-effects meta-regression framework. Data were analysed in SPSS version 28.0 (SPSS Inc., Chicago, IL, United States) and plotted in GraphPad Prism. For all statistical tests completed, a *p*- < 0.05 was assumed to be statistically significant. The inverse-variance weighting method was applied, and between-study variance was estimated using REML with Knapp-Hartung method of SE adjustment for sub-group analyses (conservative estimates due to small sample sizes).

## 3 Results

### 3.1 Characteristics of the included studies

Eighty-six studies were included, predominantly single-centre, retrospective cohorts, with biomarkers predominately measured in the serum/blood (including cord blood) (*n* = 80/84, 94%), alone or in addition to other body-fluids (CSF/urine), of neonates during hospitalisation (Figure 2). Forty-four studies assessed bilirubin exposure (Ahlfors and Parker, 2008; Ahlfors et al., 2009; Amin et al., 2016; Amin et al., 2017a; Amin et al., 2017b; Amin et al., 2024; Can et al., 2015; Chen et al., 2006; Choi et al., 2020; Dai et al., 2022; Dowley et al., 2009; Duman et al., 2004; El Houchi et al., 2017; Erdeve et al., 2018; Farouk et al., 2018; Gkoltsiou et al., 2008; Govaert et al., 2003; Hack et al., 2000; Hosono et al., 2002; Iskander et al., 2014; Jangaard et al., 2008; Kamath et al., 2024; Kang et al., 2020; Kitai et al., 2020; Kuzniewicz and Newman, 2009; Kuzniewicz et al., 2014; Lee et al., 2023; Mandour et al., 2020; Marlow et al., 2000; Martínez-Cruz et al., 2008; Matshete et al., 2024; Nickisch et al., 2009; Oh et al., 2003; Oh et al., 2010; Oyinwola et al., 2023; Saavedra and Kumar, 2018; Sharma et al., 2006; Thangavelu et al., 2019; Wickremasinghe et al., 2015; Wong et al., 2006; Xu et al., 2019; Zhang et al., 2022) and 32 evaluated infectious causes (Airede et al., 2008; Aleem et al., 2024; Alshaikh et al., 2014; Barton et al., 2014; Bashir et al., 2017; Bassler et al., 2009; Benjamin et al., 2006; Brumbaugh et al., 2022; Chen et al., 2025; Coenraad et al., 2010; Czech-Kowalska et al., 2021; da Silva et al., 2007; de Andrade et al., 2008; Doctor et al., 2001; Doneda et al., 2024; Eras et al., 2014; Fourgeaud et al., 2024; Hcini et al., 2012; Iwasaki et al., 2007; Jabłońska et al., 2025; Jung, 2020; Khairy et al., 2018; Lin et al., 2012; Medoro et al., 2024; Parlakay et al., 2023; Ronchi et al., 2020; Shimizu et al., 2019; Stoll et al., 2004; Yamaguchi et al., 2023; Yilmaz et al., 2021; Zavattoni et al., 2014; Zhou et al., 2024), and 10 examined “other”, namely—metabolic, inflammatory, respiratory, or haematological biomarkers (Chen et al., 2021; Fitzgerald et al., 2019; Jung et al., 2022; Klinger et al., 2005; Morimoto et al., 2010; Poets et al., 2015; Schmidt et al., 2013; Shim et al., 2018; Smit et al., 2013; Sriranjan et al., 2022). Overall methodological heterogeneity was high, and meta-analyses were not feasible for all outcomes. Summary biomarker study data are included in a data sheet (Supplementary material).

### 3.2 Risk-of-bias and heterogeneity

Figure 3 summarises the risk of bias across studies by category. Overall, the majority of studies were of unclear risk of bias. The number of sources with high and low risk of bias were quite even. Publications recording biomarkers of infection were more likely to have “low” risk of bias data, particularly for “population”. Key sources of methodological heterogeneity included non-uniform diagnostic criteria for hearing loss/NDI, variability in biomarker sampling timing and methodology, differences in treatments, and comorbid risk factors, and incomplete adjustment for potential confounders (e.g., prematurity, infection, hypoxia etc.).

Risk of bias and heterogeneity can also be introduced for hearing outcome measures. The majority of studies included use of hearing aid/CI/device or confirmed the degree of hearing loss. However, the was a small subsample which used AABR or UNHS results as their outcome measure, which is problematic, since a high number of false positives can be recorded from these data.

Substantial statistical heterogeneity was observed in the three biomarker categories. Cochran’s Q test was highly significant for bilirubin (*Q* = 44,447; df = 20; *p* < 0.001), infection (*Q* = 77.5, df = 19, *p* < 0.001) and “other” (*Q* = 69.91, df = 11, *p* < 0.001) demonstrating that the observed variation in effect sizes exceeds that expected by chance alone and the *I*^2^ statistic approached 100% (bilirubin), 81% (infection), 97% (other). The estimated between-study variance was large [τ^2^ = 0.42 (bilirubin), 0.6 (infection); 0.34(other)], further supporting marked dispersion of effect sizes.

Despite heterogeneity, the overall pooled effect was statistically significant for each category of biomarker; [bilirubin: overall effect size = 2.39, 95% CI (1.75–3.22), z = 5.59, *p* < 0.001; infection: effect size = 2.79 (2.1–3.74), z = 6.83, *p* < .001 and “other”: effect size = 1.90 (1.28–2.77), z = 3.26. *p* = 0.001], indicating an association in the direction of the summary estimate. For pooled estimates, emphasis was placed on the direction of effect and subgroup-specific findings rather than the magnitude of the summary estimate. Consequently, we conducted subgroup analysis and moderator analysis.

Publication bias was not statistically significant for bilirubin (intercept = 0.254, *p* = 0.19), nor infection (intercept = -0.025, *p* = 0.96) for data included in meta-analyses. This would indicate that small-study effects or funnel plot asymmetry were not problematic (Supplementary Figure 1). However, considering the large heterogeneity and small sample sizes one has to be cautious when interpreting these data.

### 3.3 Bilirubin—subgroup analyses

For a full summary of records reporting bilirubin (see Supplementary datasheet). TSB was the most commonly reported marker (*n* = 44), UB was rarely reported in addition to TSB (*n* = 6), and indirect proxies such as bilirubin/albumin (B/A) ratios (*n* = 3, plus one case report) were variably reported and often non-standardised.

Of the 44 records reporting on bilirubin, *n* = 14 sources (27 studies from the 14 sources) reported odds ratios (Figure 4). Most ORs lay to the right of the null line, indicating higher odds of hearing loss and/or NDI among infants with higher bilirubin levels. The degree of heterogeneity was high, with point estimates ranging from modest (slightly elevated odds) to strongly elevated (ORs substantially > 1). Confidence intervals were frequently wide, particularly for B/A ratios reflecting small sample sizes and differing outcome and biomarker measures. Population characteristics also varied, most were term or near-term infants (Amin et al., 2016; Amin et al., 2017a; Amin et al., 2017b; Oyinwola et al., 2023; Thangavelu et al., 2019). Marlow et al. (2000) included children born 26–31 weeks, similarly to Zhang et al. (2022) and Martínez-Cruz et al. (2008) who included children 28–43 weeks and 26–40 weeks, respectively (Martínez-Cruz et al., 2008). Hack et al. (2000), Sampath et al. (2005), and Oh et al. (2003) included low birthweight infants (<1,000 g; 23–33 weeks).

The magnitude of association varied across studies. However, the direction of effect was largely uniform, favouring bilirubin as a predictor of hearing-related pathology. Individual group effect sizes were variable; effect sizes were significant for UB [effect size = 2.34 (95% CI = 1.14, 4.81) *t* = 4.8, *p* = 0.008], HB requiring ET [effect size = 7.17 (3.00, 18.17) *t* = 3.78, *p* = 0.011] and HB plus additional illness [effect size = 4.35 (1.34, 14.15) *t* = 5.35, *p* = 0.033]. Pooled effect sizes showed borderline non-significance for TSB (*p* = 0.056) and could not be calculated for B/A ratio (*n* = 2) as both sources used the same population. Note, only emboldened data (and enlarged symbols in the plots) were included when calculating the pooled effect size to reduce the potential for statistical dependency into the meta-analytic data set (multiplicity when reporting data from the same participants), see Methods for more detailed explanation.

#### 3.3.1 Unbound bilirubin as a key predictor of hearing loss

While guidelines often rely on TSB ≥ 20–25 mg/dL (342–428 μmol/L) to indicate significant risk, the concentration that is truly neurotoxic physiologically depends on whether the bilirubin is unbound. Additionally, the individual effect size calculation from OR data sources indicated that UB is a potential candidate biomarker for hearing loss. The strongest data comes from the prospective studies by Amin et al. (2017b). Mean peak UB concentration in infants who developed hearing loss in the form of chronic AT (ANSD and hearing loss) was significantly higher (4.74 μg/dL) than in infants without AT (1.44 μg/dL) (Amin et al., 2017b). For every unit increase in peak UB (μg/dL), the odds of chronic AT increased significantly (OR = 2.41, 95% CI: 1.43–4.07, *P* = 0.001) (Amin et al., 2017b). The probability of abnormal automated auditory brainstem response (AABR) results increased significantly with increasing UB concentrations but *not* with increasing TSB concentrations (Amin et al., 2016). In subgroup analyses, when TSB was < 25 mg/dL, the peak UB was significantly associated with chronic AT (OR = 3.31, 95% CI: 1.09–10.0, *P* = 0.03). The association of increasing UB concentrations with poorer outcomes in extremely low birthweight (ELBW) infants holds true regardless of clinical status (stable or unstable), whereas TSB concentrations were only directly associated with adverse outcomes in unstable infants (Oh et al., 2010). CBE is suggested to occur in full-term newborns at UB concentrations of ≥ 2.0 μg/dL (Hosono et al., 2002). A peak UB concentration of ≥ 2.4 μg/dL was identified as having high sensitivity (0.80) and specificity (0.80) for predicting ANSD in late preterm and term infants with severe HB (Hosono et al., 2002).

#### 3.3.2 Effect of treatment for HB on hearing

Despite the small number of studies included, a significant effect size was found for hearing loss following treatment for HB with exchange transfusion (ET) (Figure 4). However, association does not equal causation, and it is difficult to separate ET effects from the effects of severe and/or prolonged HB (when ET would be implemented). In one cohort of neonates with severe HB [defined as peak TSB > 20 mg/dL or 342 μmol/L (Kemper et al., 2022)], those treated with ET *and* phototherapy showed a significantly higher incidence rate of ANSD at follow-up (11.97% in phototherapy group cf 11.57% in ET group) compared with those classified as having non-severe HB (Xu et al., 2019). Despite treatment, some long-term deficits persisted. A study of infants (≥ 28 weeks of gestational age) who underwent ET for severe HB reported 64 cases (13.9%) with poor outcomes (defined as death, cerebral palsy (CP), psychomotor delay, NDI, or hearing loss, *n* = 37 HL) at 12 months, and this incidence was positively related to peak TSB concentration (Zhang et al., 2022).

Phototherapy has documented physiological associations (retinopathy of prematurity, ROP; patent ductus arteriosus (PDA), cell oxidation) and debated developmental associations, particularly when used in high-risk preterm infants (Wang et al., 2021). Multiple long-term follow-up studies suggest that for term infants and even certain low birth weight cohorts, treatment of bilirubin does not necessarily lead to adverse neurodevelopmental outcomes. A multicentre randomised controlled study concluded that phototherapy effectively controlled neonatal HB without adverse outcome in a long-term follow-up study—assessing children at 6 years (Oh et al., 2003). Similarly, another study noted no abnormal effects of phototherapy in their cohort (Wong et al., 2006). Successful intensive phototherapy without ET in otherwise healthy term newborn infants with marked HB (20–24 mg/dL) might not increase the risk of bilirubin brain injury—where none of the infants had hearing loss, developmental delay, or abnormal neurological findings at 2–6 years of age following phototherapy (Duman et al., 2004). Oh et al. (2010) conducted a large trial comparing aggressive vs. conservative phototherapy in ELBW infants yet found no statistically significant interaction between the phototherapy treatment (aggressive or conservative) and bilirubin variables regarding poor outcomes (death or NDI).

#### 3.3.3 Risk of hearing loss relative to TSB concentration

While the association between TSB and hearing loss did not reach statistical significance for the eight studies included in the estimate, the direction and magnitude of effect were consistent with related bilirubin biomarkers, suggesting a potentially meaningful biological signal. Given the known neurotoxicity of bilirubin and the vulnerability of the neonatal brain, even modest elevations in risk may have important clinical implications.

CBE occurred in 8.5% of infants who reached high HB concentrations (TSB ≥ 30 mg/dL) (Wickremasinghe et al., 2015). However, CBE in this group only occurred when there were additional risk factors (such as prematurity, G6PD deficiency, or sepsis) and TSB concentrations were at least 15 mg/dL above AAP exchange levels (Kuzniewicz et al., 2014). There is case report evidence suggesting that even moderate HB ( ≥ 17.0 mg/dL) may increase the future risk of milder neurological sequelae, such as developmental delays, attention deficit hyperactivity disorder, and autism (Saavedra and Kumar, 2018). It could be that peak serum bilirubin (PSB) concentrations are important to measure or the number of times a child is exposed to high PSB. In ELBW infants, PSB concentrations were directly correlated with death or NDI, hearing loss (defined in the study as hearing loss that would benefit from hearing aids), and low Psychomotor Developmental Index (PDI < 70) (Oh et al., 2003). Another factor is that some studies only assessed hearing screening failure (which has a high false positive rate) or early diagnostic ABRs (recovery can occur following treatment or neuronal maturation), or hearing loss was only documented if child wore hearing aids/cochlear implants—potentially missing mild-moderate hearing losses or NICU infants lost to follow-up.

#### 3.3.4 Effect of HB and additional illness on hearing loss

Effect size was statistically significant when additional illness accompanied HB (Figure 4). The odds increased by 1.138 (95% CI: 1.00–1.30, *p* ≤ 0.05) for every unit increase in PSB for children who were prescribed amplification (Oh et al., 2003). Marlow showed that if peak bilirubin concentrations coexisted with netilmicin use the OR was 14.2, 95% CI: 1.8–114 (Marlow et al., 2000). If acidosis occurred when bilirubin concentrations were over 200 μmol/L (11.7 mg/dL) the OR was 8.0 (95% CI: 0.9–71.6) (Marlow et al., 2000). In one treated (ET) cohort, sepsis increased the odds of a poor outcome (including hearing loss) by approximately 4.35 times (95% CI: 2.013–9.409; *p* < 0.001) (Zhang et al., 2022).

#### 3.3.5 Specificity and sensitivity of bilirubin biomarkers for hearing loss and/or adverse outcomes

ROC analyses demonstrate the predictive superiority of UB, which yields a significantly larger AUC of 0.866–0.92 than TSB and exhibits enhanced specificity (up to 0.80 at 80% sensitivity) across diverse neonatal populations (Amin et al., 2016). Furthermore, the integration of clinical assessment tools enhances diagnostic precision, with a BIND score (clinical assessment) (Johnson et al., 1999) of 4 demonstrating 97.4% sensitivity and 87.3% specificity for poor neurologic outcomes, particularly when used in conjunction with the bilirubin-albumin molar ratio to achieve a combined sensitivity of 100% (El Houchi et al., 2017; Kang et al., 2020; Table 1).

**TABLE 1:** Predictive performance characteristics of bilirubin biomarkers for neurotoxicity outcomes and hearing loss.

Biomarker/score (source)	Predictive metric	Outcome	Sensitivity	Specificity	AUC
BIND score (El Houchi et al., 2017)	Score ≥ 4	Poor neurologic outcome (residual deficit at 3–5 months)	97.4%	87.3%	N/A
BIND score (El Houchi et al., 2017)	Score ≥ 4	Long-term hearing loss (bilateral REFER on AABR)	92.6%	87.3%	N/A
Bilirubin/albumin ratio (Kang et al., 2020)	≥ 8.9 mg/g	Adverse outcomes (death, hearing loss, cerebral palsy, CP) in infants with acute bilirubin encephalopathy (ABE)	92.0%	90.2%	0.895 (for B/A alone)
BIND Score (Kang et al., 2020)	Score ≥ 6	Adverse outcomes (death, hearing loss, CP) in infants with ABE	84%	77.1%	0.839 (for BIND alone)
Combined Metrics (Kang et al., 2020)	B/A ≥ 8.9 mg/dL AND BIND ≥ 6	Adverse outcomes in infants with ABE	100.0%	81.3%	0.957 (for combined model)
Peak Total serum bilirubin (TSB) (Zhang et al., 2022)	≥ 26.5 mg/dL (452.9 umol/L)	Poor Outcomes. Post-treatment—ET (death, CP, hearing loss)	81.3%	75.1%	0.837
Peak unbound bilirubin (UB) (Amin et al., 2017b)	≥ 2.4 ug/dL	Acute Auditory Neuropathy Spectrum Disorder (ANSD)	80% (0.29–0.99)	80% (0.64–0.91)	0.92

BIND score (Bilirubin-Induced Neurological Dysfunction) is a 0–9 point clinical tool used to assess the severity of Acute Bilirubin Encephalopathy (ABE) in newborns. It measures neurological status, muscle tone, and cry characteristics to determine the risk of bilirubin-induced neurological impairment. Categories: 0: Normal newborn; 1–3 (Subtle/Mild ABE): Suggests early, usually reversible, bilirubin neurotoxicity; 4–6 (Moderate ABE): Indicates intermediate involvement that may be reversed with prompt treatment; 7–9 (Advanced ABE): Indicates severe neurological involvement, with high risk for neurological impairment or death.

### 3.4 Infection-related biomarkers

The 32 infective conditions studies focused primarily on ELBW or very preterm infants susceptible to late-onset infections, and newborns diagnosed with congenital infections (CMV, Toxoplasmosis, Zika). They largely reported severe complications like NDI, hearing loss, and CP. Several studies investigated bacterial meningitis and factors which indicate a poorer outcome such as high CSF protein or seizures. Common pathogens include Group B streptococci and *Escherichia coli*. Multiple reports highlight the morbidity and mortality associated with fungal infections (most reported, Invasive Candidiasis—IC), and *Coagulase Negative Staphylococcu*s (CoNS) sepsis in ELBW infants.

Twenty-eight studies reported ORs for hearing loss and/or NDI (Figure 5). Most ORs lay to the right of the null line, indicating higher odds of hearing loss and/or NDI among infants with higher infection levels. Effect sizes were statistically significant for each of the subgroups (except for sepsis), cCMV, fungal infection and meningitis despite the conservative statistical constraints. Heterogeneity was high. This could reflect the number of potential infective disease pathogens included in the infection category and/or variability in hearing outcome measures. CIs were wide, associated with smaller sample sizes (Lin et al., 2012) but more consistent in size than in the bilirubin data. The magnitude of association varied across studies. However, the direction of effect was largely uniform, particularly favouring meningitis, CMV, and fungal infection as potential predictors of hearing loss. The largest effect in the sepsis group was found by Kamath et al. (2024) and one could argue this data could be recategorised to the meningitis sub-group as *streptococcus pneumoniae* is the leading cause of bacterial meningitis

#### 3.4.1 Meningitis

Effect sizes were significant for biomarkers of meningitis [effect size = 3.71 (1.21, 11.36), *t* = 3.01, *p* = 0.03]. Neonatal bacterial meningitis is a severe disease associated with high morbidity and mortality. Hearing loss found during hospitalisation is itself a factor predicting poor prognosis in neonatal bacterial meningitis (Lin et al., 2012). Rates varied significantly based on the definition of loss. Chen et al. (2025) reported that 52% of children had some level of hearing loss—most had mild-moderate hearing loss, 4.9–6.5% had severe or profound loss (Chen et al., 2025). Aleem et al. (2024) found that 12% of infants failed their hearing screening before discharge. Other studies reported permanent hearing loss in 8% of extremely preterm infants and 5–12.2% in very low birthweight groups (Doctor et al., 2001). In a large analysis of bacterial meningitis cases in Southern China, multivariate analysis identified movement disorder and *Streptococcus pneumoniae* or *Escherichia coli* in blood bacterial culture as independent risk factors for severe and profound hearing impairment (Chen et al., 2025). When comparing gram-negative and gram-positive proven sepsis cases, Yilmaz et al. (2021) found no difference in hearing loss rates between the groups—however, hearing loss was only determined by ABR screening.

#### 3.4.2 Specific pathogens associated with hearing loss

##### 3.4.2.1 Coagulase-negative staphylococcus

CoNS is identified as a major cause of meningitis in extremely preterm infants, though its impact on hearing is often described as less severe than other pathogens. It has been shown that CoNS often presents with a sparsity of typical inflammatory markers, which can complicate the assessment of risk of hearing loss (Doctor et al., 2001). In many cases of CoNS meningitis, the CSF leucocyte count, protein, and glucose levels remain within normal ranges (Doctor et al., 2001). Infants with late-onset meningitis (the majority caused by CoNS) had the highest incidence of hearing loss (8%) compared to those with bloodstream infections only (5%) or no infection (3%) (Brumbaugh et al., 2022). In a retrospective study there was no significant difference in the incidence of deafness (defined as hearing loss requiring amplification) between infants with CoNS sepsis (5%) and the control group (2.2%) (Alshaikh et al., 2013). However, when the definition was expanded to include milder forms of hearing loss (not requiring amplification), infants exposed to CoNS sepsis were at a significantly higher risk (9.6% vs. 3.1%, *p* = 0.01) (Alshaikh et al., 2013). This suggests that while CoNS infection itself may not serve as a powerful specific predictor of severe deafness, the associated systemic inflammation could contribute to milder hearing loss. Of concern is that many studies only assessed severe-profound hearing loss or use of hearing aids/cochlear implants as indicative of hearing loss.

##### 3.4.2.2 Invasive fungal infections (candidiasis)

Fungal infections were significantly associated with hearing loss [pooled effect size = 1.99 (1.43–2.77), *p* = 0.012] (Figure 5). Govaert et al. (2003) showed IC to be significantly associated with the rate of hearing loss and NDI compared to uninfected matched controls in very low birthweight infants (Barton et al., 2014). In ELBW infants, an abnormal hearing test was statistically more likely in the fungal infection group (OR = 2.38 in univariate analysis, OR = 1.93 in multivariate analysis) (Jung, 2020).

##### 3.4.2.3 Congenital viral infections

Studies of congenital infections, particularly cCMV, identified specific viral and inflammatory proteins in blood/plasma that correlated strongly with long-term hearing and neurological outcomes (Figure 5). There was a significant pooled effect for CMV [effect size = 7.17 (3.0–18.17), *p* = 0.025]. CMV DNA load in neonatal blood or urine correlated with a risk for SNHL, but the correlation between blood viral load and the severity or presence of neurological symptoms, including hearing loss, was inconclusive (Yamaguchi et al., 2023). However, high CMV DNAemia was suggested to predict CMV sequelae in asymptomatic congenitally infected newborns whose mothers had primary infection during pregnancy (Forner et al., 2015). Infection with the CMV gN4c genotype showed a possible association with SNHL in newborns with cCMV (Jabłońska et al., 2025).

#### 3.4.3 Novel plasma protein biomarkers of hearing loss

A recent proteomic analysis of neonatal plasma samples identified several candidate protein biomarkers for neurological complications, including hearing loss, in cCMV-infected patients (Yamaguchi et al., 2023). Plasma fms-related receptor tyrosine kinase 4 (FLT4) levels were found to be significantly higher in patients with cCMV-related symptoms, including isolated SNHL. This suggests that FLT4 may be a novel diagnostic biomarker for SNHL in cCMV infection during the neonatal period (Yamaguchi et al., 2023). They further showed that peptidylprolyl isomerase A (PPIA) levels were significantly higher in patients with neuroimaging abnormalities. Although primarily linked to CNS structural damage, PPIA is a systemic inflammatory marker, suggesting its elevation reflects broader neurological compromise. In a small cohort, the persistence of PD-1+ CD8 + T cells (markers suggesting T cell exhaustion) over the first year of life was observed among infants with progressive SNHL after cCMV infection, compared to those with nonprogressive SNHL and normal hearing (Medoro et al., 2024).

#### 3.4.4 Other congenital infections (toxoplasmosis and Zika virus)

The included studies did not specify plasma or serum biomarkers for predicting SNHL in toxoplasmosis and often had very small cohorts of children with hearing loss. Children often appeared healthy at birth and were only identified through neonatal screening programs, such as those detecting IgM anti-T. gondii (de Andrade et al., 2008). Kuzniewicz et al. (2014) indicated that without screening, toxoplasmosis-related hearing loss is often categorised as “unknown”—their study found that 4/19 children with congenital toxoplasmosis had hearing loss (de Andrade et al., 2008). Researchers of the Zika-virus exposed cohort reported a higher rate of hearing loss (2/15, 13.3%) at 2 years of age among infants with congenital Zika infection (Hcini et al., 2012).

#### 3.4.5 Cerebrospinal fluid markers

Biomarkers measured in CSF often reflect CNS infection severity which is directly linked to hearing loss risk. In symptomatic cCMV infection, high CSF β2-microglobulin (b2-m) levels (> 7.9 mg/L) showed high specificity for identifying infants at increased risk for moderate-severe adverse outcome (Alarcon et al., 2013). The detection of CMV DNA in CSF (CSF-CMV-PCR) was considered a marker of severe CNS injury and a predictor of subsequent outcomes, including severe SNHL (Czech-Kowalska et al., 2021). In neonatal bacterial meningitis, initial CSF protein > 2.0 g/L and initial CSF glucose < 1.0 mmol/L were risk factors for severe and profound hearing loss (Chen et al., 2025). The most precise figures for sensitivity and specificity related specifically to markers of CNS involvement in symptomatic cCMV infection (Alarcon et al., 2013). This predictive accuracy was maximised when a biochemical marker is combined with neuroimaging findings or a clinical score (Alarcon et al., 2013; Fourgeaud et al., 2024; Table 2). High specificity but low sensitivity indicates the test (CSF -microglobulin (CSF -m) +) rarely misdiagnoses healthy neonates but could miss neonates with cCMV.

**TABLE 2:** Predictive performance for biomarkers of infection, congenital CMV.

Biomarker/Score (source)	Outcome	Sensitivity	Specificity
CSF -microglobulin (CSF -m)—cCMV (Alarcon et al., 2013)	Unfavourable outcome (death or moderate/severe disability) with symptomatic cCMV	69%	100%
CSF -m or Adjusted Microcephaly—cCMV (Alarcon et al., 2013)	Unfavourable outcome	82%	100%
CSF -m or Neuroimaging Score (Grade 2–3)—cCMV (Alarcon et al., 2013)	Unfavourable outcome	61%	100%
Neuroimaging Score (Grade 2–3)—cCMV (Fourgeaud et al., 2024)	Absence of neurological sequelae at 2 years of age	69%	98%
Neonatal Predictive Model (Hearing, Platelet, Cranial US) (Zavattoni et al., 2014)	Symptomatic cCMV infection at birth	61.5%	45.5%

CSF-microglobulin (CSF-m), specifically beta2-microglobulin (β2-m), is a cerebrospinal fluid biomarker used to assess central nervous system (CNS) involvement and predict neurological damage in infants with symptomatic congenital CMV (cCMV) infection. Elevated CSF-m levels indicate active neuroinflammation and correlate with neuroimaging abnormalities.

### 3.5 Other biomarkers of hearing loss

Other biomarkers were categorised as either inflammation, metabolic stress, multi-organ dysfunction, or functional iron status. The ten sources included biochemical indicators of risk for neonatal hearing loss or impairment relating to evidence of severe systemic stress or inflammation reflected by metabolic derangements (abnormal glucose, low cord pH), multi-organ injury (elevated creatinine; aspartate aminotransferase, AST; alanine aminotransferase, ALT), and acute-phase reactants (elevated IL-6 and CRP). Shim et al. (2018) reported that high concentrations of Complement C3a in the umbilical cord may indicate a protective mechanism against hearing screen failure (OR = 0.875, *p* = 0.023) (Shim et al., 2018). However, only two confirmed cases of SNHL were recorded (Shim et al., 2018). In the proteomic analysis conducted by Yamaguchi et al. (2023) complement C3 was investigated as a candidate plasma biomarker for neurological complications in cCMV (Yamaguchi et al., 2023). Save for respiratory biomarkers, there was insufficient of each subtype to quantify with respect to effect size.

#### 3.5.1 Respiratory

In extremely preterm infants, prolonged intermittent hypoxemia episodes defined as oxygen saturation < 80% lasting approximately 1 min or longer ( ≥ 6 consecutive 10-s values) were strongly associated with severe hearing loss (requiring hearing aids or cochlear implants), as part of the composite outcome of late death or disability (Poets et al., 2015). Shorter hypoxemic episodes were not significantly associated with adverse outcomes. Episodes of severe hyperoxaemia (PaO_2_ > 26.6 kPa or 200 mm Hg) or severe hypocapnia (PaCO_2_ < 2.6 kPa or 20 mm Hg) during the first 2 hours of life in HIE infants were individually and synergistically associated with adverse outcome, which included deafness (Klinger et al., 2005).

All studies (*n* = 3 sources) in the respiratory subcategory (Figure 6) had an OR with wide CI but predominantly OR > 1 indicative of respiratory events (pulse oximetry data) causing hearing loss and/or NDI. The seven studies included in the respiratory OR plot derive from three studies (Figure 6). Overall, the effect size for “other” was significant. However, in subgroup analyses only three respiratory data were included, and the effect size was not statistically significant. It could be argued that only two studies should be included, as the data reported by Bassler et al. (2009) were collected prospectively on the cohort reported by Schmidt et al. (2013). When this study was omitted the effect size was also not significant.

#### 3.5.2 Inflammatory and immune response biomarkers (CRP, IL-6, C3a)

Pooled effect sizes were not calculated for the inflammatory and immune response biomarkers as insufficient data were available. It was shown that in preterm neonates, high CRP levels ( > 5 mg/L) in the immediate postnatal period were significantly associated with UNHS test failure (Shim et al., 2018), possibly reflecting perinatal inflammatory or infective insults. In NICU-treated infants with ABR abnormalities, an elevated CRP concentration was found to be significantly associated with an elevation of the ABR threshold in childhood, potentially predicting hearing deterioration (Chen et al., 2025). The same study found that blood CRP levels > 50 mg/L (5 mg/dL) were independent predictors of hearing loss, which is itself associated with permanent neurosensory sequelae (Chen et al., 2025). A single study concluded that elevated concentrations of the inflammatory cytokine IL-6 in umbilical cord plasma may contribute to the risk of UNHS failure (Shim et al., 2018); the best cutoff value identified for predicting failure was 3.37 pg/mL.

Proteomic analysis identified other differentially expressed proteins in cord blood associated with adverse neurodevelopmental outcomes including deafness in monoamniotic twins (Jung et al., 2022). They showed that immunoglobulin (Ig)-gamma-4 chain C region, Apolipoprotein E, and Alpha-fetoprotein were all upregulated. Whereas there was downregulation in: Ig-lambda chain V region 4A, Ig-heavy variable 3, Ig-kappa chain C region, Ig-mu chain C region, Complement C1q, Ceruloplasmin, and Ig-lambda chain V-I region (Jung et al., 2022). In subsequent validation, Jung et al. (2017) showed that the cord blood concentration of ceruloplasmin was significantly lower in neonates with adverse neurodevelopmental outcomes. Ceruloplasmin is involved in copper and iron metabolism and antioxidant activity; low levels may indicate neurodegeneration.

#### 3.5.3 Metabolic and multi-organ dysfunction in relation to hearing loss

Several metabolic measurements reflecting systemic distress and multi-organ failure have been linked to hearing impairment, particularly in infants with hypoxic ischemic encephalopathy (HIE). Abnormal initial blood glucose (<2.6 mmol/L or > 10 mmol/L) was significantly associated with hearing loss in term infants treated with therapeutic hypothermia for HIE (Fitzgerald et al., 2019). Specifically, early hypoglycaemia (<46.8 mg/dL or < 2.6 mmol/L) in the first postnatal hour was significantly associated with permanent hearing loss in therapeutically cooled infants (Fitzgerald et al., 2019). In addition, high creatinine concentrations and raised liver transaminases were observed in infants who developed hearing loss (Fitzgerald et al., 2019). They showed that raised creatinine concentrations, indicative of multi-organ dysfunction (acute kidney injury), were statistically significantly higher in infants who developed hearing loss post-HIE treatment on Day 1 (*p* = 0.0172), Day 2 (*p* = 0.0198), and Day 3 (*p* = 0.0409). Raised liver transaminases, indicative of multi-organ dysfunction (liver injury), were significantly associated with hearing loss in HIE infants (AST, *p* = 0.0012 and ALT, *p* = 0.0037) (Fitzgerald et al., 2019). A low cord blood pH was identified as significantly associated with hearing loss in infants treated with therapeutic hypothermia for HIE (Smit et al., 2013). A higher rate of hearing loss (*n* = 11/78) among the patients with moderate-to-severe neonatal HIE was observed (χ^2^ (1, *n* = 78) = 5.39, *p* = 0.020) than those with mild HIE (Chen et al., 2021). Furthermore, a higher percentage of neonates had hearing loss than those without HIE (14.1% vs. 0.87%; *p* < 0.001). The patients who exhibited hearing loss had significantly higher lactate (104.8 ± 51.0 vs. 71.4 ± 48.4; U = 181, *p* = 0.032) and serum glucose (159.5 ± 86.1 vs. 112.1 ± 62.3; U = 166, *p* = 0.036) concentrations than those without hearing loss (Chen et al., 2021).

## 4 Discussion

### 4.1 Summary of findings

Biochemical data provided by neonates admitted to the NICU offer an optimal research advantage as concentrations of biomarkers in serum, urine, and CFS are measured routinely from the moment they are admitted.

This systematic review synthesises evidence from 86 sources on biochemical, infectious, inflammatory, metabolic, and physiological biomarkers associated with neonatal hearing loss and related neurodevelopmental sequelae in high-risk neonatal populations. Despite substantial methodological and statistical heterogeneity, a consistent direction of effect was observed across bilirubin-, infection-, and other-related biomarker categories, supporting a biologically plausible association between systemic neonatal illness and hearing loss.

Among bilirubin-related biomarkers, UB emerged as the most specific and predictive biomarker for hearing loss, outperforming TSB in both effect size and diagnostic accuracy. These findings reinforce experimental and clinical evidence that neurotoxicity is driven by the unbound fraction rather than total bilirubin burden, highlighting limitations of current TSB-based thresholds. PSB and number of times an infant is exposed to PSB could also be an important factor. The increased risk observed when HB co-occurred with additional illness underscores the multifactorial nature of bilirubin-induced neurotoxicity and the importance of clinical context.

Infectious biomarkers demonstrated robust associations with hearing loss, particularly for meningitis, congenital CMV, and invasive fungal infections. These findings are consistent with known mechanisms of inflammation-mediated cochlear and neural injury. Emerging proteomic and CSF biomarkers, especially in congenital CMV, showed high specificity for severe outcomes, suggesting potential for future risk stratification, although validation remains limited.

“Other” biomarkers reflecting hypoxia, inflammation, and multi-organ dysfunction further support the concept that hearing loss in neonates is often a downstream manifestation of systemic injury rather than an isolated pathology. However, many studies relied on hearing screening failure rather than confirmed permanent hearing loss, limiting interpretability.

Overall, while pooled estimates were statistically significant, the high heterogeneity necessitates cautious interpretation.

### 4.2 Implications for research

Future research should prioritise multicentre studies using standardised hearing outcomes, repeated biomarker measurements, and multivariable adjustment. Integration of biochemical markers with clinical scoring systems may offer the greatest potential for improving early identification and prevention of neonatal hearing loss.

From a translational perspective, quantitative biomarkers may offer greater utility for risk stratification and threshold-based clinical decision-making by enabling assessment of dose-response relationships, whereas binary clinical exposures primarily identify at-risk populations without defining actionable biological treatment thresholds.

NICU populations present substantial barriers to behavioural and hardware-based testing, so future studies should test the feasibility of serial, minimally invasive sampling to support continuous monitoring—even during physiological instability, ventilation, or periods where middle-ear status compromises OAE/ABR accuracy. A further key direction involves identifying modifiable biological pathways that precede hearing loss. Markers of inflammation, hypoxia–ischaemia, oxidative stress, or ototoxic medication exposure may enable targeted risk reduction strategies, such as antibiotic stewardship, optimisation of oxygenation/perfusion, or trials of anti-inflammatory and/or neuroprotective therapies. Hearing loss in high-risk neonates often co-occurs with broader neurological vulnerability so future work should examine whether composite biomarker profiles can predict both auditory and neurodevelopmental outcomes, improving parental counselling and timing of interventions.

Ultimately, high-quality cohort studies are needed to support precision-medicine approaches. This includes defining reference ranges, understanding developmental trajectories of biomarkers, validating diagnostic accuracy, and integrating biomarker data with clinical risk factors. Combining biomarker panels, audiologic data, and NICU exposures may allow stratified follow-up pathways, earlier intervention, and prevention of irreversible auditory–neural injury.

#### 4.2.1 Bilirubin and neurotoxicity

The review confirms bilirubin as the most extensively studied biomarker for hearing loss. While TSB remains the cornerstone of clinical decision-making, its poor specificity limits its utility as a standalone predictor of neurotoxicity. Across studies, UB showed more consistent and biologically plausible associations with both acute and chronic AT, including ANSD and SNHL, and particularly in late preterm and term infants with severe HB. Importantly, the data reinforce that bilirubin neurotoxicity is context-dependent: extremely preterm and clinically unstable infants may experience neurotoxicity at TSB levels far below conventional treatment thresholds, whereas healthy term infants generally tolerate much higher concentrations. These findings support a shift away from rigid TSB cut-offs toward risk-stratified approaches incorporating binding capacity, gestational age, and comorbidities.

#### 4.2.2 Infection and inflammation

In the investigation of infection as a biomarker for hearing loss, the utility of traditional blood culture results is limited, primarily indicating the presence of a generalised systemic inflammatory risk. Infectious diseases, particularly neonatal meningitis, invasive fungal infections, and congenital viral infections, were consistently associated with hearing loss and adverse neurodevelopmental outcomes. Traditional microbiological markers primarily reflect disease severity rather than serving as specific predictors of hearing loss.

Bacterial meningitis was associated with the worst outcomes, trending toward increasing rates of significant NDI (32.0%) compared to late-onset culture-positive bloodstream infection (22.9%) and non-infected infants (15.0%). While meningitis itself was highly predictive of SNHL and overall poor prognosis, identification of specific highly virulent pathogens like *Streptococcus pneumoniae* or *Escherichia coli* were independent risk factors for severe and profound hearing loss in neonatal bacterial meningitis. Fungal infections (IC) were also critically implicated, with affected ELBW children exhibiting drastically increased risks of deafness (OR 5.37) and death or NDI (up to 79%). In contrast, CoNS sepsis appears less strongly associated with severe deafness requiring amplification, though it increases the risk of the milder form of neurosensory deafness (9.6% vs. 3.1% in unexposed infants) and cognitive delay (Adjusted OR was 2.23). Emerging evidence from cCMV infection suggests that targeted plasma and CSF biomarkers (e.g., FLT4, β2-microglobulin, CSF CMV DNA) may improve early risk stratification for hearing loss, especially in infants who are asymptomatic at birth (Alarcon et al., 2013; Czech-Kowalska et al., 2021; Yamaguchi et al., 2023). However, these findings are based on small cohorts and require external validation.

Systemic inflammation emerged as a common mechanistic pathway linking diverse exposures to hearing loss. Elevated inflammatory markers (CRP, IL-6) were repeatedly associated with UNHS failure, progressive hearing loss, and composite neurodevelopmental disability. These markers likely reflect a broader inflammatory milieu that potentiates neural and cochlear vulnerability, rather than direct ototoxicity.

#### 4.2.3 Other biomarkers

The evidence base for hearing loss biomarkers is strengthened by studies focusing on markers reflecting critical insults in vulnerable neonates, successfully associating measures of systemic inflammation (e.g., high early postnatal CRP and cord plasma IL-6), metabolic stress (e.g., abnormal initial blood glucose), and multi-organ dysfunction (e.g., raised creatinine and liver transaminases) with hearing loss or UNHS failure. Prospective collection of variables—gentamicin trough concentrations—allowed some studies to identify specific pharmacological risks. It should be noted that in the general population prevalence of the mitochondrial m.1555A > G mutation which confer marked susceptibility to aminoglycoside induced ototoxicity is approximately 0.2–0.26% (United Kingdom) (Rahman et al., 2012). This is an important example of gene-environmental interaction and a target for future personalised monitoring. Despite the low prevalence it has potential significance in the NICU population. Although NICU-specific prevalence data are lacking, studies of hearing loss cohorts suggest enrichment of the mutation among infants with permanent sensorineural hearing loss, particularly following aminoglycoside exposure.

Markers of metabolic derangement, organ failure, and disordered oxygenation were strongly associated with hearing loss in high-risk populations, particularly infants with HIE and extremely preterm infants. Prolonged hypoxaemia, severe abnormalities in blood gases, hypoglycaemia, and renal or hepatic dysfunction were consistent predictors of adverse outcomes, reinforcing the concept that hearing loss is often a manifestation of global neurological injury rather than an isolated deficit.

However, there are major limitations including small sample sizes and low event rates for permanent hearing loss in the high-risk HIE cohorts, constraining statistical power for multivariate analysis. These include reliance on UNHS failure as a less specific surrogate outcome measure due to the low prevalence of confirmed hearing loss, and the use of retrospective or post hoc designs for physiological markers like prolonged intermittent hypoxemia or severe hyperoxaemia which establish associations rather than definitive causation of auditory injury.

In a subset of studies, hearing screening referral was used as the primary outcome measure; as screening failure does not necessarily equate to confirmed permanent hearing loss, these associations can reflect immaturity, technical issues etc. rather than definitively established hearing loss.

Interpretation of UNHS referral outcomes should be undertaken cautiously, as screening results do not confirm permanent hearing loss. Evidence indicates substantial false-positive rates (Colella-Santos et al., 2014). In England, the positive predictive value of NICU screening referrals is approximately 10.9% for permanent childhood hearing loss, meaning most referrals do not result in confirmed impairment (Wood et al., 2015)). Although higher diagnostic yields have been reported more recently elsewhere (e.g., 24.4% in Belgium) (Verstappen et al., 2023), variability in predictive value across settings limits the reliability of UNHS referral status as a definitive outcome measure.

### 4.3 Strengths and limitations of the evidence

A key strength of this review is the comprehensive inclusion of diverse biomarker domains, reflecting the multifactorial nature of neonatal hearing loss. However, the certainty of evidence is limited by substantial methodological heterogeneity, inconsistent biomarker measurement, variable definitions and timing of biomarker collections, and ill-defined hearing outcome data. Meta-analysis was frequently precluded by these factors. Moreover, many studies did not adequately adjust for confounders such as prematurity, sepsis, or concurrent treatment exposures, limiting causal inference.

Several included studies reported hearing loss as part of composite neurological outcomes, most commonly NDI. While this reflects the reporting structure of many neonatal cohorts, it limits the ability to infer hearing-specific predictive precision for certain biomarkers. Associations derived from composite outcomes may reflect broader neurological vulnerability rather than auditory pathway specific pathology. Accordingly, these findings should be interpreted as indicators of global neurodevelopmental risk that may include hearing loss, rather than as biomarkers validated specifically for auditory outcomes. Future studies should prioritise disaggregated hearing endpoints and standardised audiological phenotyping to enable pathway specific validation.

The majority of studies were judged to have an unclear or high risk of bias (Figure 3), irrespective of the biomarker studied. Across biomarker research the predominating reason for high risk of bias in population were small sample sizes (particularly for the hearing loss groups) and few control groups were reported. Demographic data were often well reported but there was rarely control for multiple confounders for example, chronological age, gestational age, birth weight, sex—which are risk factors for hearing loss.

The overall certainty of evidence was low to moderate due to observational designs, inconsistent biomarker measurement, variable outcome definitions, and incomplete adjustment for confounding. While bilirubin, especially UB and markers of severe infection or systemic instability appear plausible predictors of neonatal hearing loss, substantial heterogeneity limits precise risk quantification.

Methodologically, most studies were retrospective analyses of hospital assay data which are an important source of highly relevant clinical data. However, there was little mention of the timings or number of samples taken, repeatability, missing data, reference data, type of assays used across hospital sites, or whether methodology changed over time.

In terms of analyses—corrections for multiple comparisons were rarely implemented and identification of a true biomarker for hearing loss was difficult to ascertain as it was rare for concentrations of biomarkers to be included, therefore in most cases sensitivity/specificity and cut-off thresholds could not be ascertained.

### 4.4 Research priority

The standardisation of research methodologies in individualised biomarker medicine is essential to reduce the significant statistical and clinical heterogeneity that currently complicates evidence synthesis in systematic reviews. The following recommendations outline how future primary studies can be improved to facilitate more robust comparisons and meta-analyses.

#### 4.4.1 Standardisation of exposure variables and units

The sources demonstrate a significant lack of uniformity in the biomarkers used to quantify biomarker concentration.

#### 4.4.2 Uniform definitions for neurodevelopmental impairment and hearing loss

The definition of “poor outcome” or NDI varies widely between cohorts, often including a disparate mix of motor, cognitive, and sensory deficits. Comparative accuracy would be greatly enhanced if studies utilised a standardizsed composite endpoint. There were substantial discrepancies in the methodology used to define hearing loss, ranging from simple AABR to diagnostic ABRs with varying decibel (dB) thresholds. Future research should specify the diagnostic threshold used for hearing loss not screening results, as benchmarks currently fluctuate widely across different studies, whereas others only indicate use of hearing aids/cochlear implants as an indication of hearing loss.

#### 4.4.3 Categorisation by clinical stability and gestational age

Research into ELBW infants indicates that clinical status functions as a potent effect modifier, meaning, for example that TSB levels may only be predictive of injury in “unstable” neonates. To improve comparability, studies should explicitly include additional risk factors or classify infants as “stable” or “unstable” using objective criteria such as blood pH below a commonly agreed value, positive blood cultures for sepsis, or the requirement for mechanical ventilation and pressor support. Additionally, cohorts should be stratified by narrow gestational age bands (e.g., < 28 weeks, 28–34 weeks, 34–36 weeks, and ≥ 37 weeks) because the physiological threshold for neuronal injury shifts dynamically according to developmental maturity. Alternatively, a matched control group should be included that is matched on gestational age, birth weight, and sex.

#### 4.4.4 Alignment of assessment chronology

The timing of audiological and neurological assessments relative to the biomarker peak is a critical source of methodological heterogeneity. Systematic reviews require that researchers provide long-term follow-up data (at least up to 18–24 months) to distinguish transient changes from permanent neurological sequelae.

#### 4.4.5 Systematic reporting of co-occurring risk factors

The synergy between biomarkers and clinical comorbidities, such as bilirubin and sepsis, metabolic acidosis, and G6PD deficiency, often determines the risk of injury at lower biomarker thresholds. Future studies should provide detailed frequencies and adjusted ORs for these co-occurring risk factors, as their presence necessitates a downward adjustment of intervention thresholds. Universal screening for G6PD deficiency within the study population is also recommended, as it is a leading cause of hazardous HB but is frequently under-assessed.

#### 4.4.6 Comprehensive statistical reporting for meta-analysis

To facilitate the calculation of the statistics and other measures of consistency in future systematic reviews, primary researchers must report more than just a *p*-value and control for multiple comparisons. Easy comparison requires the inclusion of: Means and standard deviations (SD) for continuous variables [or medians (IQRs) for non-parametric data]. Full effect sizes with 95% confidence intervals (CI) for all outcome associations. ROC analysis, specifically reporting the AUC, sensitivity, and specificity for specific biomarker concentration cut-off thresholds.

## 5 Conclusion

Neonatal hearing loss is most consistently linked to two modifiable biological risk factors: HB and infection. Among bilirubin measures, UB showed a stronger and more reliable association with hearing loss than total serum bilirubin, predicting both acute abnormalities and long-term outcomes such as SNHL and ANSD, especially in term and extremely low birth weight infants. Treatment of HB, including phototherapy and ET, can rapidly reduce bilirubin though persistent deficits still occur in a subset of infants, particularly when other vulnerabilities (prematurity, sepsis) are present.

Evidence for infection-related biomarkers remains limited: traditional markers only indirectly reflect risk, while emerging proteomic studies, notably in congenital CMV, have identified promising candidates (e.g., FLT4, PPIA). However, these findings are early, heterogeneous, and not yet ready for clinical use. Elevated FLT4 suggests vascular and lymphatic pathway involvement in hearing loss, while increased PPIA reflects systemic inflammatory activation associated with structural brain abnormalities. Sustained T-cell exhaustion phenotypes further implicate dysregulated adaptive immunity in progressive hearing loss. Collectively, these markers highlight a biologically plausible, blood-based strategy for early risk stratification and targeted monitoring. However, translation into clinical pathways requires rigorous, adequately powered, multi-centre external validation with harmonised phenotyping, longitudinal follow-up, and standardised assay platforms to ensure reproducibility, generalisability, and clinically meaningful decision thresholds.

## Data Availability

The original contributions presented in the study are included in the article/Supplementary material, further inquiries can be directed to the corresponding author.
