# Supplementary material for: Biological markers of hearing loss in neonates admitted to the neonatal intensive care unit: a systematic review and meta-analysis
Source: Front Neurosci. 2026 Mar 31;20:1796635. doi: 10.3389/fnins.2026.1796635 (PMC13076293; doi:10.3389/fnins.2026.1796635)
Supplement: Supplementary file 2 [file Data_Sheet_2.docx]

**Fig. 1 Supplementary Material: Funnel Plots (data included in meta-analyses for pooled effect size, Fig. 4 and 5, selected emboldened data)**

1.
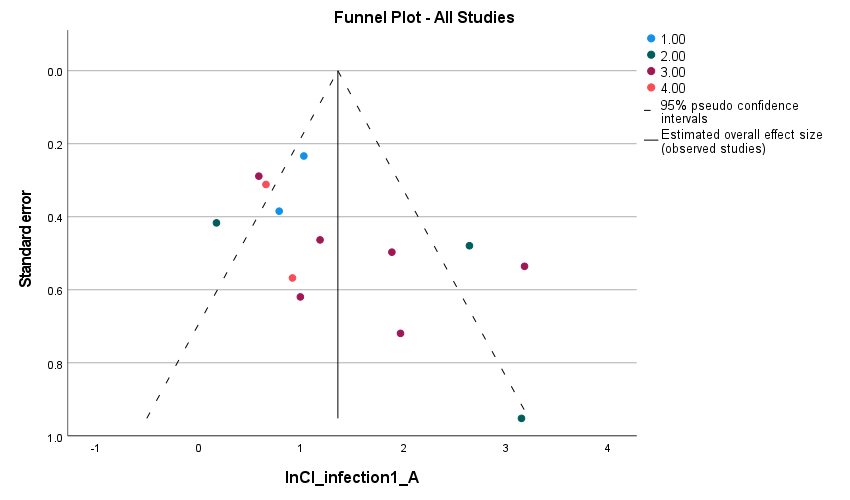

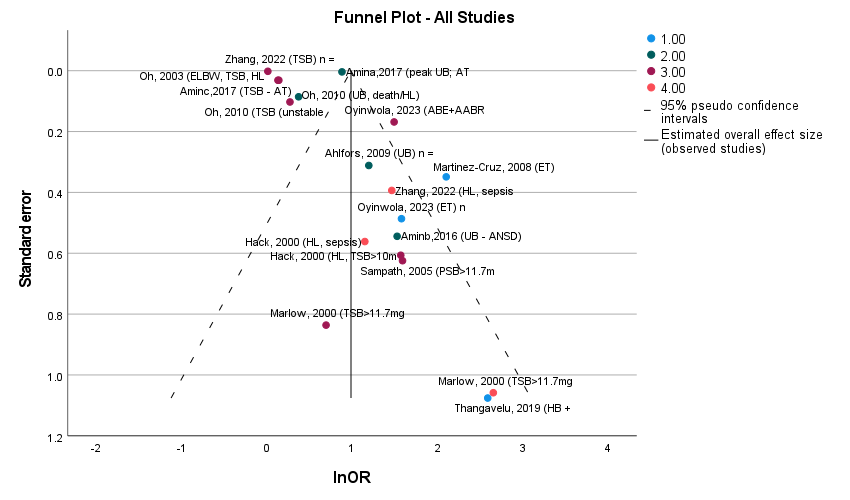
**Bilirubin-related biomarkers** **B. Infection-related biomarkers**
